# Supplementary material for: TBL1X/TBL1XR1 govern β-cell identity through a PAX6-containing gene regulatory network
Source: Nat Commun. 2026 Apr 23;17:3736. doi: 10.1038/s41467-026-72077-5 (PMC13102947; doi:10.1038/s41467-026-72077-5)
Supplement: Supplementary file 1 — Supplementary Information [file 41467_2026_72077_MOESM1_ESM.pdf]

**Supplemental Figure 1:**

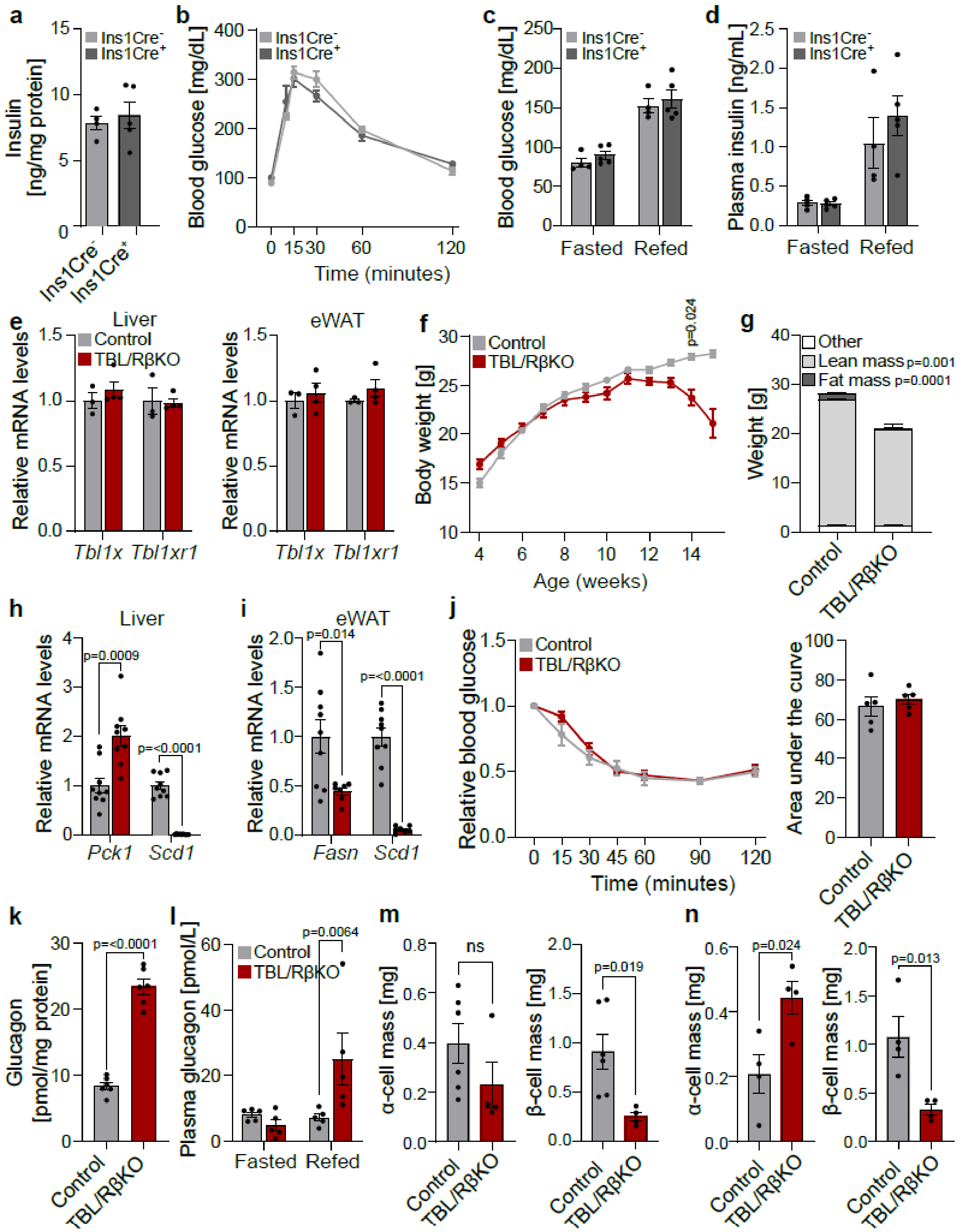


**a**, Total pancreas insulin content normalized to protein levels in Ins1Cre+ (n=5) and Ins1Cre- (n=4) and mice. **b**, Blood glucose levels during an intraperitoneal glucose tolerance test in Ins1Cre+ (n=5) and Ins1Cre- (n=4) mice. **c** and **d**, 16 h fasting and 2 h refeeding blood glucose (**c**) and plasma insulin (**d**) levels in Ins1Cre+ (n=5) and Ins1Cre- (n=4) and mice. **e**, Relative *Tbl1x* and *Tblr1xr1* mRNA expression in liver and gonadal white adipose tissue (eWAT) in TBL/RβKO (n=3) and control (n=4) mice at 5 weeks of age determined using qPCR. **f**, Body weight over time in TBL/RβKO (n=6) and control (n=6) mice. **g**, Body composition determined by Echo-MRI in TBL/RβKO (n=6) and control (n=6) mice at 15 weeks of age. **h** and **i**, Relative

mRNA expression of catabolic genes in liver (**h**) and gonadal white adipose tissue (**i**) in TBL/RβKO and control mice at the age of 20 weeks determined using qPCR. Liver control *Pck1*: n=9, control *Scd1*: n=8. TBL/RβKO *Pck1*, *Scd1*: n=9. eWAT control: n=9, TBL/RβKO: n=7. **j**, Relative blood glucose changes during an intraperitoneal insulin tolerance test and the area under the curve in TBL/RβKO (n=5) and control (n=5) mice at 11 weeks of age. **k**, Total pancreas glucagon content normalized to protein levels in TBL/RβKO (n=6) and control (n=6) mice at the age of 15 weeks. **l**, 16 h fasting and 2 h refeeding plasma glucagon levels in in TBL/RβKO (n=5) and control (n=5) mice at the age of 15 weeks. **m** and **n**, α- and β-cell mass in TBL/RβKO (n=4) and control (n=6) mice at 5 weeks of age (**m**) and in TBL/RβKO (n=4) and control (n=4) mice at 20 weeks of age (**n**). Data are represented as means ± SEM. The following statistical tests were applied: Two-sided student's t-test (**a**, **e**, **g**, **h**, **i**, **j** – area under the curve, **k**, **m**, **n**), 2-way ANOVA with uncorrected Fisher's LSD *post hoc* test (**c**, **d**, **l**), and 2-way ANOVA with Šidák's multiple comparison *post hoc* test (**b**, **f**, **j** – time course). ns = no significance.

**e-l**, Gray color indicates control mice and red color indicates TBL/RβKO mice.

**Supplemental Figure 2:**

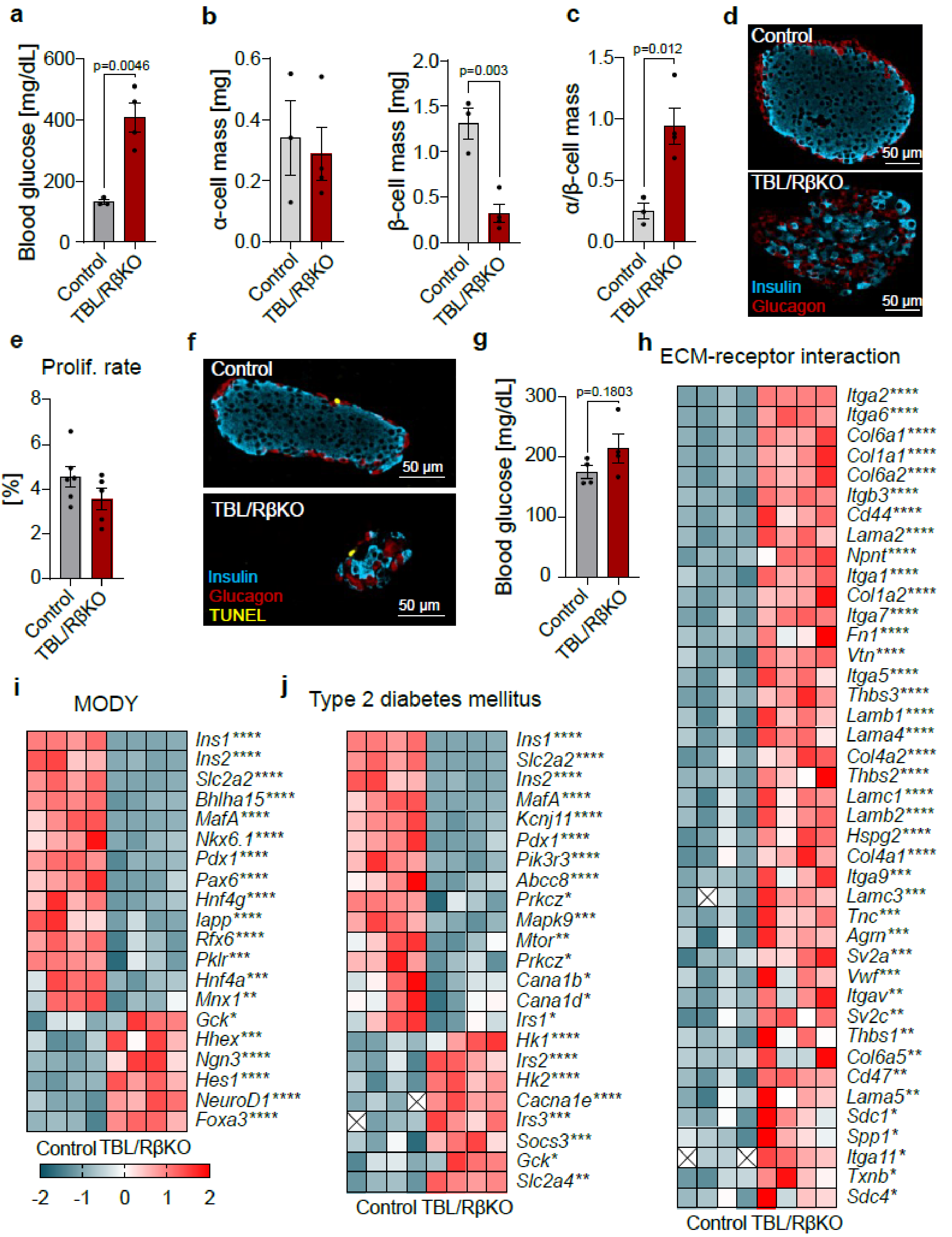


**a**, Blood glucose levels of 20 week old female TBL/RβKO (n=3) and control (n=4) mice. **b**, Pancreatic α- (left) and β-cell mass (right) of female TBL/RβKO (n=3) and control (n=4) mice at 20 weeks of age. **c**, α/β-cell mass ratio of pancreatic islets from female TBL/RβKO (n=3) and control (n=4) mice at 20 weeks of age. **d**, Representative immunofluorescent staining of insulin+ (blue, β-cells) and glucagon+ (red, α-cells) cells of paraffin embedded pancreas from female TBL/RβKO and control mice at 20 weeks of age. **e**, Proliferative rate of pancreatic β-cells in TBL/RβKO (n=5) and control (n=6) mice at the age of 5 weeks determined *via* co-

staining of insulin$^+$ and BrdU$^+$ cells. **f**, Representative immunofluorescent insulin$^+$ (blue, β-cells), glucagon$^+$ (red, α-cells), and TUNEL$^+$ (yellow, apoptotic events) staining of paraffin embedded pancreas of TBL/RβKO and control mice. Only one TUNEL$^+$ event was detected within an islet after analyzing 134 TBL/RβKO and 119 control islets, indicating that apoptosis does not account for reduced β-cell mass upon TBL/R1 knockout. **g**, Blood glucose levels of TBL/RβKO (n=4) and control (n=4) mice upon islet isolation used for bulk RNA-sequencing. **h-j**, Heatmap of differentially expressed genes identified *via* bulk RNA-sequencing using islets from TBL/RβKO and control mice associated with the KEGG Pathways ECM-receptor interaction (**d**), MODY (**e**), and type 2 diabetes mellitus (**f**). Each box represents one mouse. Color indicates a z-score for TBL/RβKO vs. control islets. **a-c**, **e**, **g** are represented as means ± SEM. Each dot represents one mouse. The following statistical test was applied: Two-sided student's t-test (**a-c**, **e**, **g**). *p < 0.05, **p < 0.01, ***p < 0.001, ****p < 0.0001.

**Supplemental Figure 3:**

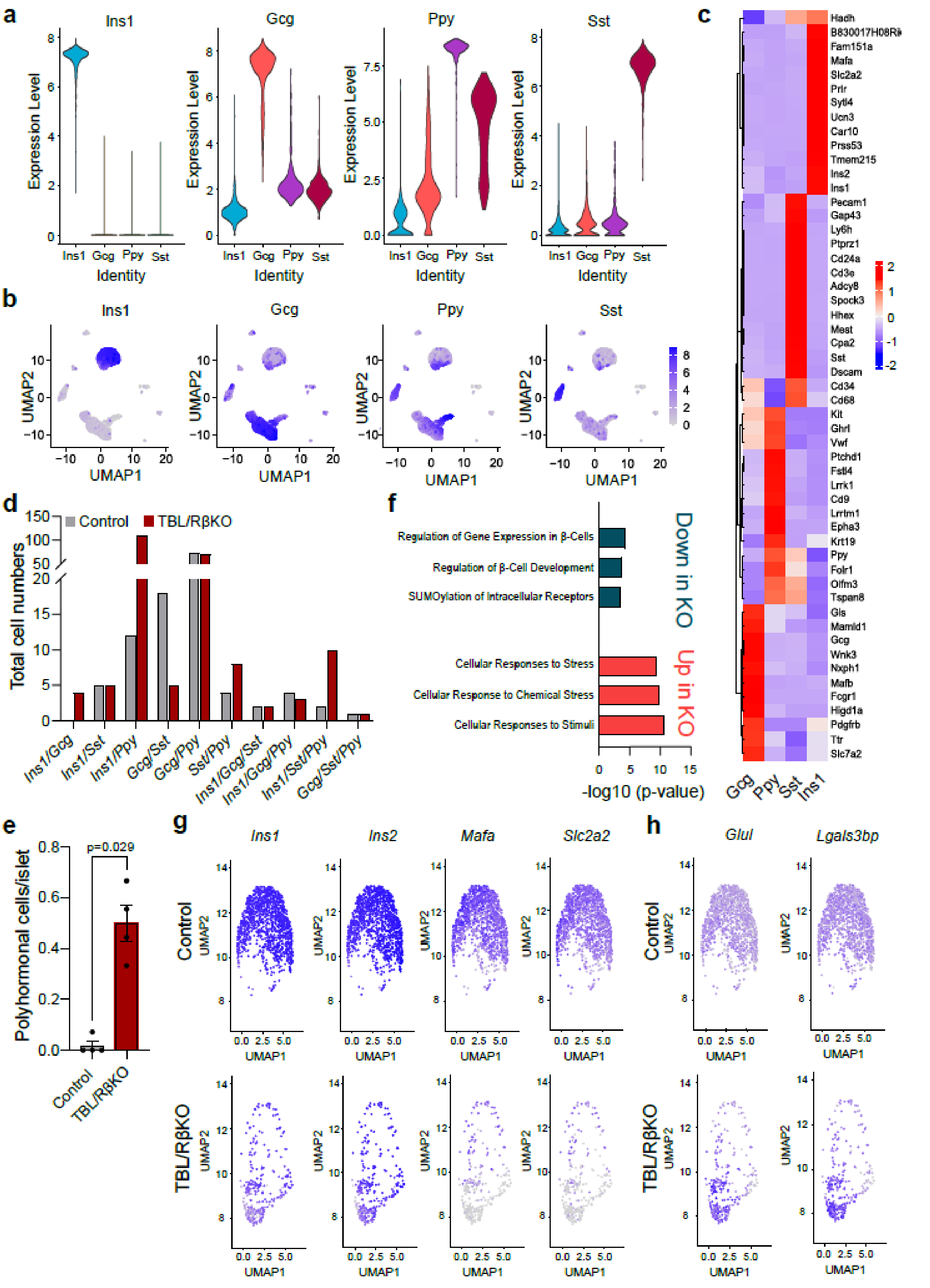


**a**, Violin plots showing the expression of *Ins1*, *Gcg*, *Ppy*, and *Sst* in cells identified as β-cells (Ins1), α-cells (Gcg), PP-cells (Ppy) and δ-cells (Sst). **b**, UMAP plot showing clustering of the main islet hormone cell populations identified by single cell RNA-sequencing of pancreatic islets from control and TBL/RβKO mice. **c**, Heatmap showing the top 10 genes that are most

highly and specifically expressed in the indicated islet hormone cell types, determined by single-cell RNA-sequencing. **d**, Bar graph showing the relative abundance of double or triple hormone positive cells identified in the single-cell RNA-sequencing. **e**, Quantification of polyhormonal cells in islets of control and TBL/RβKO mice using immunofluorescent images. Cells, that co-stained for insulin and glucagon were determined as polyhormonal. Statistical analysis was performed using the Mann-Whitney test. *p < 0.01. **f**, Functional enrichment analysis of up- and downregulated genes in Ins1$^+$ cells identified by single-cell RNA-sequencing using Enrichr. The top 3 Reactome Pathways 2024 for the up- and downregulated genes are indicated in red and blue, respectively. The following selection criteria were used: log2 fold change > 0.5 for upregulated genes and log2 fold change < -0.5 for downregulated genes, p-adj < 0.05. **g** and **h**, UMAP plot showing the expression of selected β-cell identity (**g**) and β-cell disallowed (**h**) genes in Ins1$^+$ cells identified by single-cell RNA-sequencing of pancreatic islets from control and TBL/RβKO mice. Genes were selected from the top 10 differentially up- and downregulated genes displayed in Figure 3e.

**Supplemental Figure 4:**

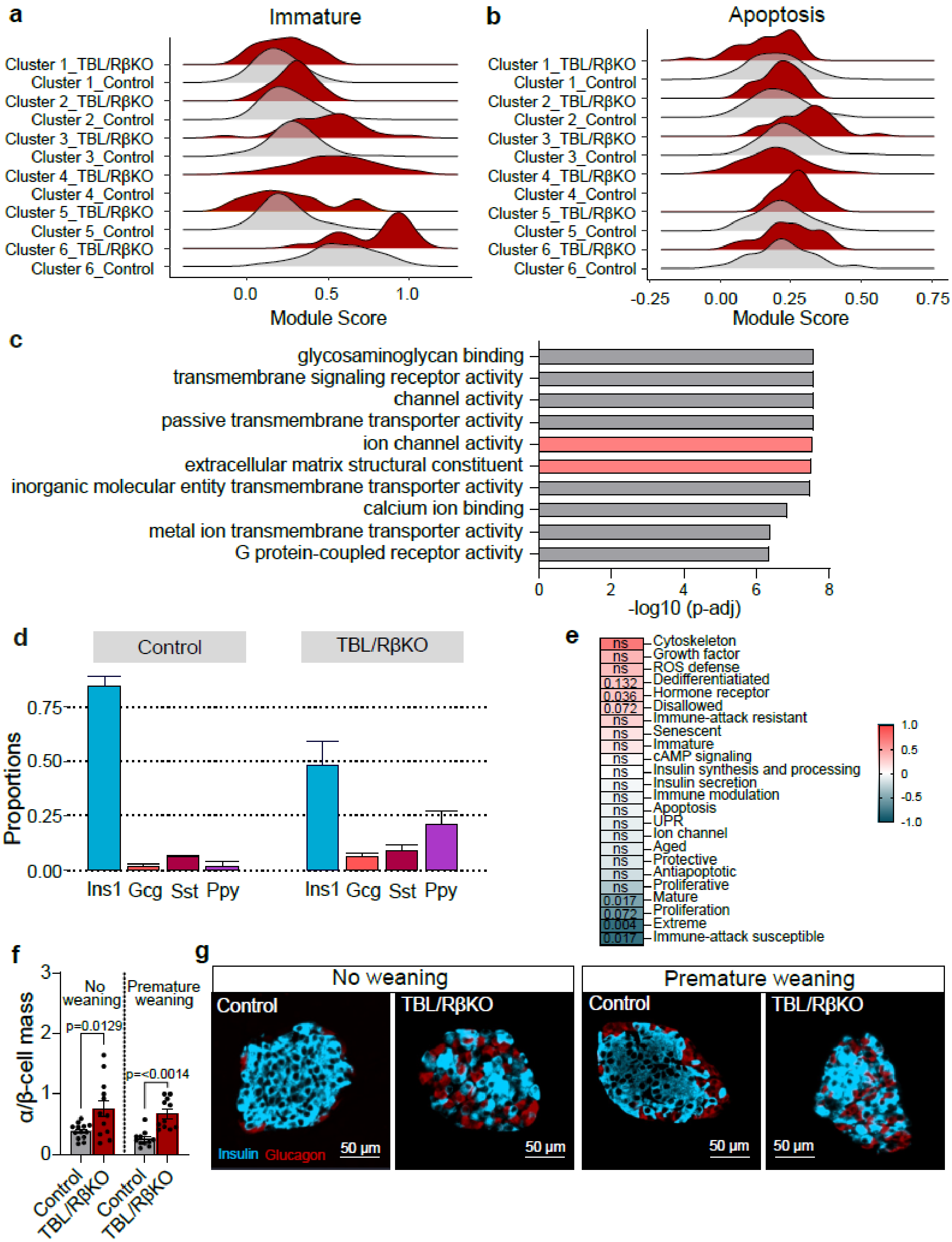


**a** and **b**, Ridgeline plot showing the abundance of genes associated with β-cell immaturity (**a**) or apoptosis (**b**) within the different clusters previously identified in Fig. 3d in islets from control and TBL/RβKO mice using single-cell RNA-sequencing. **c**, Molecular function of differentially expressed genes between TBL/RβKO and control β-cells identified using GO analysis. **d**, Proportions of the main islet hormone cell types of control and TBL/RβKO mice determined

using bulk RNA-sequencing deconvolution. **e**, Gene set variation analysis for quantification of the β-cell specific programs using deconvoluted bulk RNA-sequencing data. Displayed are the adjusted p-values. The heatmap shows the differences between control and TBL/RβKO β-cells. **f**, α/β-cell mass ratio of TBL/RβKO and control mice not (no weaning) or prematurely weaned (premature weaning). No weaning control n=13, TBL/RβKO n=12; premature weaning control and TBL/RβKO n=11. **g**, Representative immunofluorescent insulin$^+$ (blue, β-cells) and glucagon$^+$ (red, α-cells) staining of paraffin embedded pancreas from non-weaned and prematurely weaned TBL/RβKO and control mice. Each dot represents one mouse. Data in (**e**) is represented as mean ± SEM. A 2-way ANOVA with uncorrected Fisher's LSD *post hoc* test was applied.

**Supplemental Figure 5:**

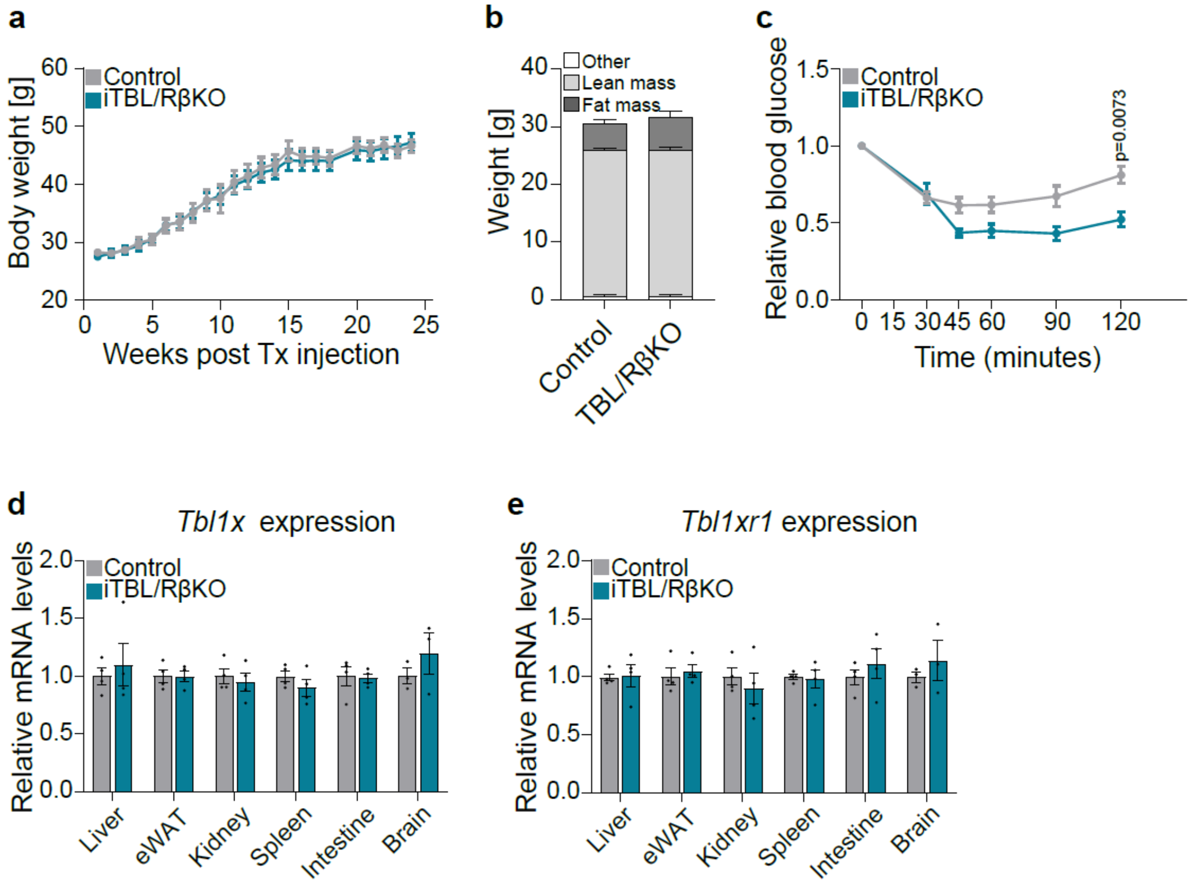


**a**, Body weight over time of iTBL/RβKO (n=9) and control (n=9) mice on HFD after tamoxifen (Tx) administration. **b**, Body composition determined by Echo-MRI of iTBL/RβKO (n=7) and control (n=8) mice on HFD, 5 weeks after tamoxifen (Tx) administration. **c**, Relative blood glucose changes during an intraperitoneal insulin tolerance test in iTBL/RβKO (n=5) and control (n=5) mice 11 weeks after tamoxifen administration. **d** and **e**, Relative *Tbl1x* and *Tbl1xr1* mRNA expression in different tissues of iTBL/RβKO (n=3) and control (n=3) mice determined using qPCR. Each dot represents one mouse. Data are represented as means ± SEM. The following statistical tests were applied: 2-way ANOVA with Šidák's multiple comparison *post hoc* test (**a**, **c**) and a two-sided student's t-test (**b**, **d**, **e**).

**Supplemental Figure 6:**

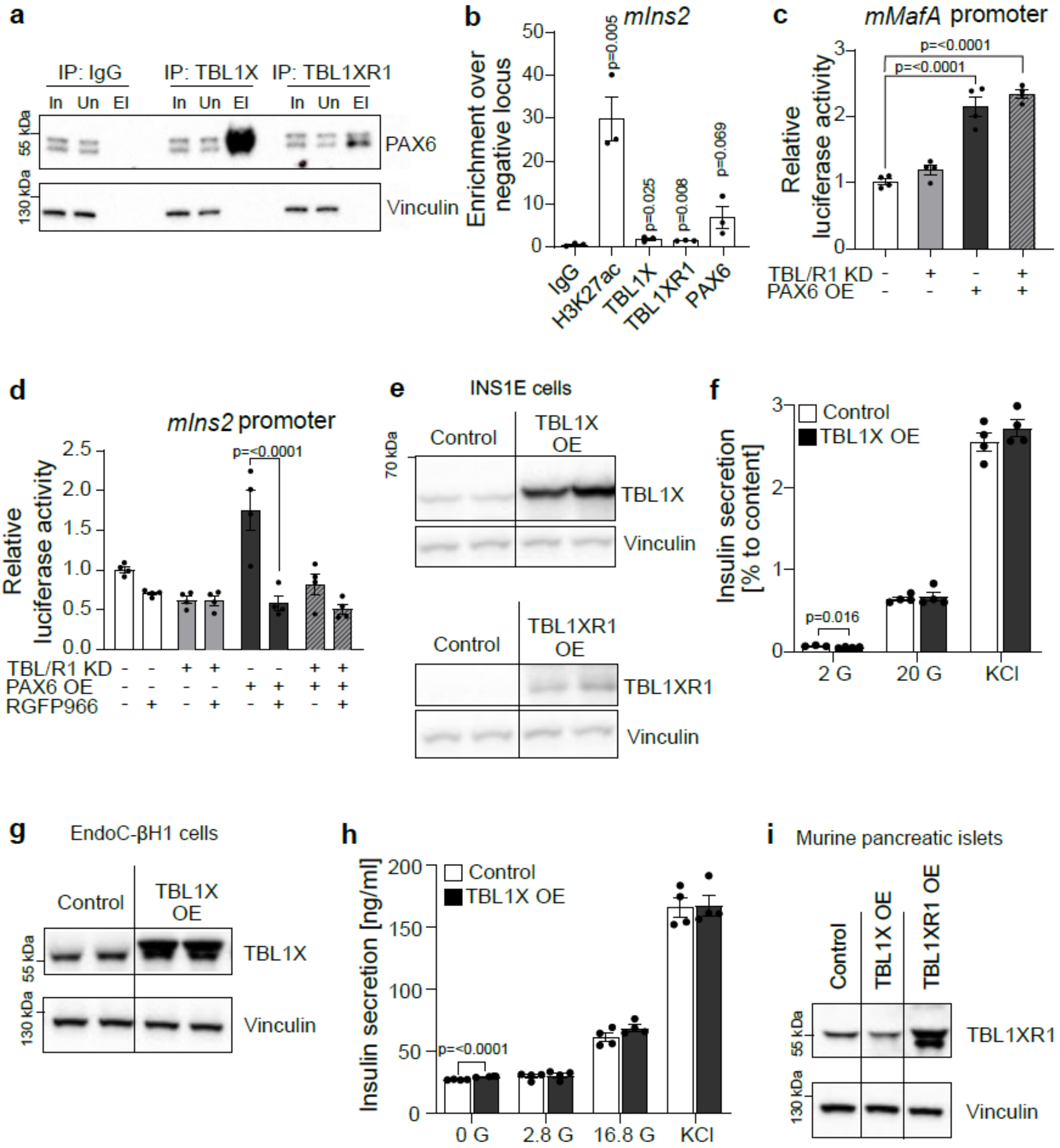


**a**, Endogenous TBL1X and TBL1XR1 was immunoprecipitated in lysates of MIN6 cells with rabbit IgG as negative control. Whole cell lysates (Input), unbound proteins (Ub) and immunoprecipitated proteins from the eluate (El) were immunoblotted using the indicated antibodies. **b**, IgG (negative control), H3K27ac (positive control), TBL1X, TBL1XR1, and PAX6 ChIP-qPCR in MIN6 cells for the murine *Ins2* locus (*mIns2*). The enrichment is calculated over the negative locus. n=3. **c**, Murine *Mafa* promoter (*mMafa*) activity in INS1E cells upon TBL/R1 knockdown or/and PAX6 overexpression. Luciferase activity was normalized to renilla luciferase. n=4. **d**, Murine *Ins2* promoter (*mIns2*) activity in INS1E cells upon TBL/R1 knockdown or/and PAX6 overexpression treated with or without the HDAC3 inhibitor RGFP966 (0.5 μM) for 24 h. Luciferase activity was normalized to renilla luciferase. n=4. **e**, TBL1X, TBL1XR1, and vinculin protein levels in INS1E cells after transfection with control or TBL1X (top panel) and TBL1XR1 (bottom panel) overexpression constructs. **f**, Insulin secretion relative to insulin content in INS1E cells upon *Tbl1x* overexpression using overexpression constructs. After glucose starvation, insulin secretion was stimulated using 2 mM glucose (2 G), 20 mM glucose (20 G), and 2 mM glucose supplemented with KCl (KCl) to promote

maximal insulin secretion. n=4. **g**, TBL1X and vinculin protein levels in EndoC-βH1 cells after transfection with control or TBL1X overexpression constructs. **h**, Insulin secretion EndoC-βH1 cells upon *Tbl1x* overexpression using overexpression constructs. After glucose starvation, insulin secretion was stimulated using 2.8 mM glucose (2.8 G), 16.8 mM glucose (16.8 G), and 2.8 mM glucose supplemented with KCl (KCl) to promote maximal insulin secretion. Basal insulin secretion was determined using 0 mM glucose. n=4. **i**, TBL1XR1 and vinculin protein levels in murine pancreatic islets upon *Tbl1xr1* overexpression using an adenovirus. Data are represented as means ± SEM. The following statistical tests were applied: Two-sided student's t-test (**b**, **f**, **h**), ordinary one-way ANOVA with Dunnett's multiple comparison post hoc test (**c**), and 2-way ANOVA with a Tukey's multiple comparison post hoc test (**d**).

**Supplemental Figure 7:**

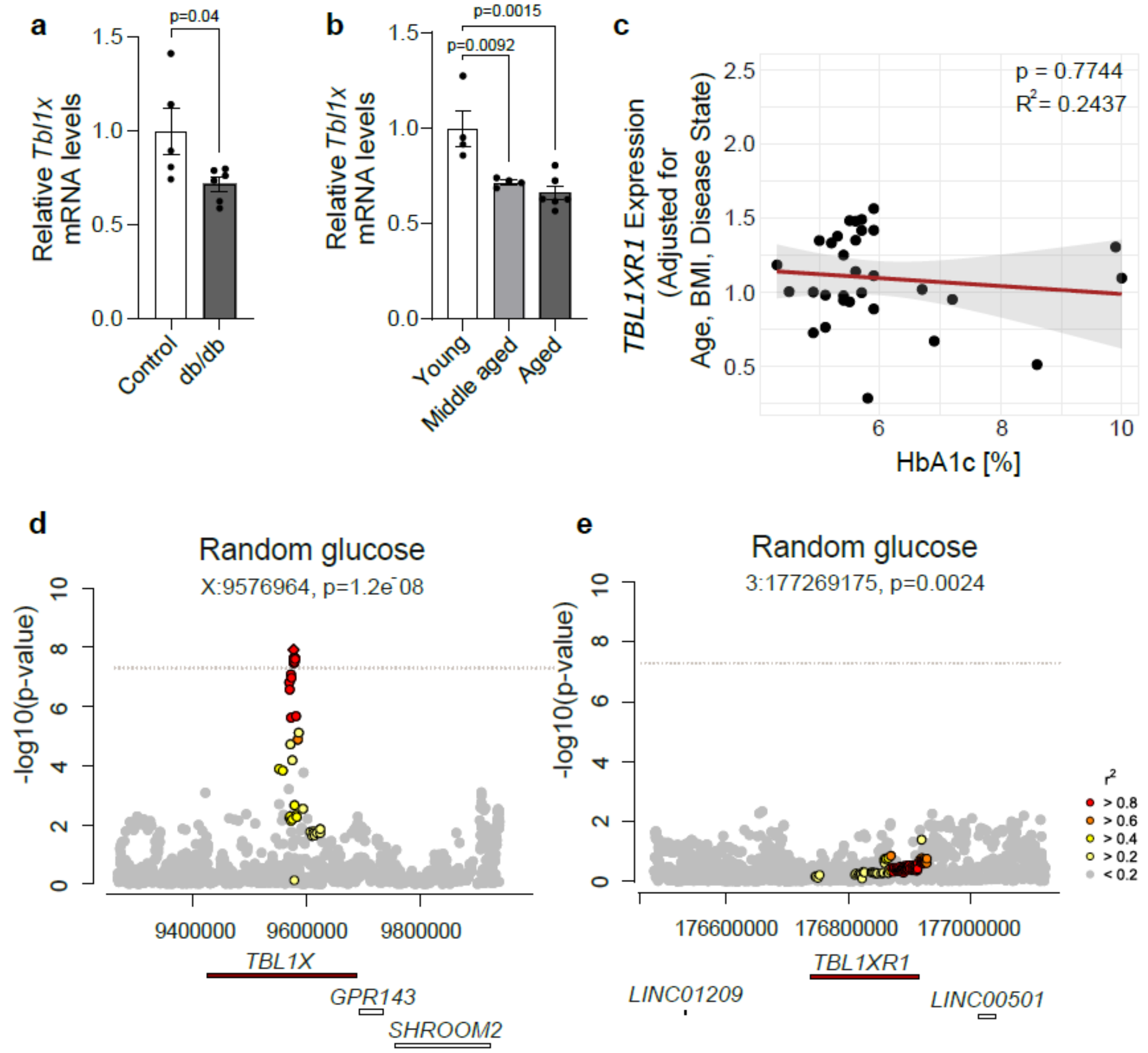


**a**, Relative *Tbl1x* mRNA levels determined by qPCR in pancreatic islets isolated from mice heterozygous (control, n=5) or homozygous (db/db, n=6) for the spontaneous diabetes mutation Lepr$^{db}$ at the age of 8 weeks. **b**, Relative *Tbl1x* mRNA levels in pancreatic islets isolated from young (10 weeks, n=4), middle aged (52 weeks, n=4), and aged mice (120 weeks, n=6), determined using qPCR. **c**, Scatter plot depicting the relationship between HbA1c [%] and relative *TBL1XR1* mRNA levels determined by qPCR, adjusted for age, body mass index (BMI), and disease state in humans. Each dot represents an individual data point. **d** and **e**, Manhattan plot showing an association between elevated random blood glucose levels and single nucleotide polymorphisms in the genetic region of *TBL1X* (**b**) and *TBL1XR1* (**c**). **a** and **b**, are represented as means ± SEM. The following statistical tests were applied: Two-sided student's t-test (**a**, **b**) and linear regression (**c**).
