# Supplementary material for: TBL1X/TBL1XR1 govern β-cell identity through a PAX6-containing gene regulatory network
Source: Nat Commun. 2026 Apr 23;17:3736. doi: 10.1038/s41467-026-72077-5 (PMC13102947; doi:10.1038/s41467-026-72077-5)
Supplement: Supplementary file 3 — Description of Additional Supplementary Files [file 41467_2026_72077_MOESM3_ESM.pdf]

Description of Additional Supplementary Files

File Name: Supplementary Data 1
Description: Beta heterogeneity markers and beta functional genes. Markers, previously identified by Hrovatin et al. 2023, used to generate gene networks presented in Figure 3f.

File Name: Supplementary Data 2
Description: Summary of the pancreatic islet donor characteristics. Islets were used for TBL1X and TBL1XR1 mRNA level determination. Full information at www.humanislets.com
