# Supplementary material for: TBL1X/TBL1XR1 govern β-cell identity through a PAX6-containing gene regulatory network
Source: Nat Commun. 2026 Apr 23;17:3736. doi: 10.1038/s41467-026-72077-5 (PMC13102947; doi:10.1038/s41467-026-72077-5)
Supplement: Supplementary file 6 — Reporting Summary [file 41467_2026_72077_MOESM6_ESM.pdf]

nature portfolio

Corresponding author(s): Rohm, Maria

Last updated by author(s): 25.03.2026

# Reporting Summary

Nature Portfolio wishes to improve the reproducibility of the work that we publish. This form provides structure for consistency and transparency in reporting. For further information on Nature Portfolio policies, see our Editorial Policies and the Editorial Policy Checklist.

## Statistics

For all statistical analyses, confirm that the following items are present in the figure legend, table legend, main text, or Methods section.

| n/a | Confirmed | |
|---|---|---|
| ☐ | ☒ | The exact sample size (*n*) for each experimental group/condition, given as a discrete number and unit of measurement |
| ☐ | ☒ | A statement on whether measurements were taken from distinct samples or whether the same sample was measured repeatedly |
| ☐ | ☒ | The statistical test(s) used AND whether they are one- or two-sided<br>*Only common tests should be described solely by name; describe more complex techniques in the Methods section.* |
| ☒ | ☐ | A description of all covariates tested |
| ☒ | ☐ | A description of any assumptions or corrections, such as tests of normality and adjustment for multiple comparisons |
| ☐ | ☒ | A full description of the statistical parameters including central tendency (e.g. means) or other basic estimates (e.g. regression coefficient) AND variation (e.g. standard deviation) or associated estimates of uncertainty (e.g. confidence intervals) |
| ☐ | ☒ | For null hypothesis testing, the test statistic (e.g. *F*, *t*, *r*) with confidence intervals, effect sizes, degrees of freedom and *P* value noted<br>*Give P values as exact values whenever suitable.* |
| ☒ | ☐ | For Bayesian analysis, information on the choice of priors and Markov chain Monte Carlo settings |
| ☒ | ☐ | For hierarchical and complex designs, identification of the appropriate level for tests and full reporting of outcomes |
| ☐ | ☒ | Estimates of effect sizes (e.g. Cohen's *d*, Pearson's *r*), indicating how they were calculated |

*Our web collection on statistics for biologists contains articles on many of the points above.*

## Software and code

Policy information about availability of computer code

| | |
|---|---|
| Data collection | Cell Ranger pipeline (version 7.1.0), DropletUtils (version 1.18.1), Seurat (version 5.1.0), “DoubletFinder” (version 2.0.4), “Scrublet” (version 0.2.2), enrichplot: R package version 1.22.0, ggraph: R package version 2.2.1.9000 |
| Data analysis | GraphPad Prism version 10.2.3, R (version 4.2.3), Mascot (version 2.4), Progenesis QI software for proteomics (Version 3.0), |

For manuscripts utilizing custom algorithms or software that are central to the research but not yet described in published literature, software must be made available to editors and reviewers. We strongly encourage code deposition in a community repository (e.g. GitHub). See the Nature Portfolio guidelines for submitting code & software for further information.

## Data

Policy information about availability of data

All manuscripts must include a data availability statement. This statement should provide the following information, where applicable:

- Accession codes, unique identifiers, or web links for publicly available datasets
- A description of any restrictions on data availability
- For clinical datasets or third party data, please ensure that the statement adheres to our policy

BBulk RNA-seq data are deposited to GEO with the dataset identifier GSE283723 (https://www.ncbi.nlm.nih.gov/geo/query/acc.cgi). scRNA-seq data are deposited to GEO with the dataset identifier GSE275357 (https://www.ncbi.nlm.nih.gov/geo/query/acc.cgi). The mass spectrometry data are deposited to the ProteomeXchange Consortium (http://proteomecentral.proteomexchange.org) via the PRIDE partner repository with the dataset identifier PXD054677 (https://www.ebi.ac.uk/pride/archive/projects/PXD054677). Source data for all figures are provided with this paper.

## Research involving human participants, their data, or biological material

Policy information about studies with human participants or human data. See also policy information about sex, gender (identity/presentation), and sexual orientation and race, ethnicity and racism.

| | |
|---|---|
| Reporting on sex and gender | Study included patients from both sexes. |
| Reporting on race, ethnicity, or other socially relevant groupings | No categorisation based on race, ethnicity or other socially relevant groupings. |
| Population characteristics | Patients' data (number of participants, body mass index, Hba1c) are summarised in Supplemental Data 2. Age and sex distribution are provided in methods section. |
| Recruitment | Human donor organs were obtained with written consent and research ethics approval of the Human Research Ethics Board (HREB) at the University of Alberta (Pro00013094, Pro00001754). |
| Ethics oversight | Human Research Ethics Board (HREB) at the University of Alberta Pro00013094, Pro00001754 |

Note that full information on the approval of the study protocol must also be provided in the manuscript.

# Field-specific reporting

Please select the one below that is the best fit for your research. If you are not sure, read the appropriate sections before making your selection.

[x] Life sciences [ ] Behavioural & social sciences [ ] Ecological, evolutionary & environmental sciences

For a reference copy of the document with all sections, see nature.com/documents/nr-reporting-summary-flat.pdf

# Life sciences study design

All studies must disclose on these points even when the disclosure is negative.

| | |
|---|---|
| Sample size | The number of replicates for the mouse studies was set based on statistical consultation for preparing animal licences (ROB-55.2-2532.Vet_02-16-136, ROB-55.2-2532.Vet_02-21-133, and ROB-55.2-2532.Vet_02-18-93). Typically, studies were performed with n=6 mice per group, with up to 2 animals/ group as reserve. In some instances, n numbers were lower, reflecting the limited availability of animals from breeding. The interactome screen was performed once with n=4 wells per condition. For cell culture experiments, n numbers were 3-5 as indicated in each figure legend. |
| Data exclusions | No data excluded |
| Replication | Key results from mouse experiments were confirmed in different independent in vivo experiments . In vitro experiments were replicated using independent cell cultures and cell passages. All attempts at replication were successful. The exact number of replications for each experiment is indicated in the respective figure legend. |
| Randomization | No randomisation. |
| Blinding | No blinding was done because knowledge of experimental conditions was required during animal handling and data collection. |

# Reporting for specific materials, systems and methods

We require information from authors about some types of materials, experimental systems and methods used in many studies. Here, indicate whether each material, system or method listed is relevant to your study. If you are not sure if a list item applies to your research, read the appropriate section before selecting a response.

### Materials & experimental systems

| n/a | Involved in the study | |
|---|---|---|
| [ ] | [x] | Antibodies |
| [ ] | [x] | Eukaryotic cell lines |
| [x] | [ ] | Palaeontology and archaeology |
| [ ] | [x] | Animals and other organisms |
| [x] | [ ] | Clinical data |
| [x] | [ ] | Dual use research of concern |
| [x] | [ ] | Plants |

### Methods

| n/a | Involved in the study | |
|---|---|---|
| [x] | [ ] | ChIP-seq |
| [x] | [ ] | Flow cytometry |
| [x] | [ ] | MRI-based neuroimaging |

## Antibodies

| | |
|---|---|
| Antibodies used | anti-Insulin, Cell Signaling Technology, Cat# 3014; RRID:AB_2126503; anti-Glucagon, Takara Bio, Cat# M182; RRID:AB_2619627, goat anti-Rabbit IgG (H+L) Cross-Adsorbed Secondary Antibody, Alexa Fluor™ 750, Thermo Fisher Scientific, Cat# A21039, RRID:AB_2535710; goat anti-Guinea Pig IgG (H+L) Highly Cross-Adsorbed Secondary Antibody, Alexa Fluor™ 647, Thermo Fisher Scientific, Cat# A21450, RRID:AB_2535867). To assess apoptosis, a terminal deoxynucleotidyl transferase dUTP nick end labeling (TUNEL) was performed using the In Situ Cell Death Detection Kit, TMR red (Roche, Cat# 12156792910), anti-TBL1X, Proteintech, Cat# 13540-1-AP, RRID: AB_2199783; anti-TBL1XR1, Novus Biologicals, Cat# NB600-270, RRID: AB_10001343; anti-PAX6, Millipore, Cat# AB2237, RRID: AB_1587367; anti-H3K27ac, Abcam, Cat# ab4729, RRID: AB_2118291; anti-normal rabbit IgG, Cell Signaling Technology, Cat# 2729, RRID: AB_1031062), Anti-Vinculin, Abcam, Cat# 129002, RRID: AB_11144129; anti-TBL1X, Abcam, Cat# ab24548, RRID: AB_2199904; anti-TBL1XR1, Santa Cruz, Cat# sc-517365; anti-PAX6, Cell Signaling Technology, Cat# 60433, RRID: AB_2797599, goat anti-rabbit HRP, BioRad, Cat# 1705046, RRID: AB_11125757; goat anti-mouse HRP, BioRad, Cat# 1706516, RRID: AB_2921252; IgG Fraction Monoclonal Mouse Anti-Rabbit IgG, light chain specific, Jackson Immunoresearch Lab, Cat# 213-032-177, RRID: AB_2339251, anti-TBL1X, Abcam, Cat# ab24548, RRID: AB_2199904; anti-TBL1XR1, Novus Biologicals, Cat# NB600-270, RRID: AB_10001343; anti-DYKDDDDK Tag, Cell Signaling Technology, Cat# 2368, RRID: AB_2217020 |
| Validation | Antibodies were not validated |

## Eukaryotic cell lines

Policy information about cell lines and Sex and Gender in Research

| | |
|---|---|
| Cell line source(s) | INS1E were from Heiko Lickert lab, RRID:CVCL_0351. MIN6 cells were from Seino lab, RRID: CVCL_4371. EndoCbH1 were shared from Novo Nordisk upon collaborative agreement, initially from Human Cell Design RRID: CVCL_L909. |
| Authentication | The cell lines have not been authenticated. |
| Mycoplasma contamination | Cell lines have undergone mycoplasma checks and tested negative. |
| Commonly misidentified lines (See ICLAC register) | No commonly misidentified cell lines were used. |

## Animals and other research organisms

Policy information about studies involving animals; ARRIVE guidelines recommended for reporting animal research, and Sex and Gender in Research

| | |
|---|---|
| Laboratory animals | All mice had C57BL/6N background. Experiments were performed starting at 8-14 weeks of age. β-cell specific TBL/R1 knockout mice were generated by breeding TBL1X and TBL1XR1 floxed mice (Taconic Artemis, Cologne, Germany) and B6(Cg)-Ins1tm1.1(cre)Thor/J mice (Jackson Laboratory). Inducible β-cell TBL/R1 knockout mice were generated by crossing TBL1X and TBL1XR1 floxed mice with mice carrying the tamoxifen-inducible Cre recombinase gene under the control of the rat insulin 2 promoter (RIP2Cre_ERT). RIP2Cre_ERT mice were kindly shared by the Ashcroft Lab. Knockout was induced by subcutaneous injection of 200 mg tamoxifen per kg body weight in corn oil. Tamoxifen-injected RIP2Cre_ERT negative littermates were used as controls. Homozygous db/db and heterozygous control mice were purchased from The Jackson Laboratory. TBL1Xfl/fl TBL1XR1fl/fl mice were used for the aging cohort. The study uses primarily male mice with key results repeated in female mice. |
| Wild animals | Study did not involve wild animals. |
| Reporting on sex | The following is reported as a limitation of the study: All data shown was generated using male mice. However, a subset of experiments was also performed in female mice showing that also female TBL/RβKO mice develop hyperglycemia. Moreover, while α-cell mass remained unchanged β-cell mass was significantly reduced determined by immunofluorescent staining of in paraffin embedded pancreas in female TBL/RβKO mice resulting in an elevated α/β-cell mass ratio. Lastly, abnormal features of islet cell distribution were also observed in female mice with α-cells scattered across the islet, as previously shown in male mice. |
| Field-collected samples | The study did not involve samples collected from the field. |
| Ethics oversight | Animal handling and experimentation were approved and performed in accordance with the guidelines from the European Union directives and the state ethics committees and government of Upper Bavaria (Germany). |

Note that full information on the approval of the study protocol must also be provided in the manuscript.

## Plants

| | |
|---|---|
| Seed stocks | *Report on the source of all seed stocks or other plant material used. If applicable, state the seed stock centre and catalogue number. If plant specimens were collected from the field, describe the collection location, date and sampling procedures.* |
| Novel plant genotypes | *Describe the methods by which all novel plant genotypes were produced. This includes those generated by transgenic approaches, gene editing, chemical/radiation-based mutagenesis and hybridization. For transgenic lines, describe the transformation method, the number of independent lines analyzed and the generation upon which experiments were performed. For gene-edited lines, describe the editor used, the endogenous sequence targeted for editing, the targeting guide RNA sequence (if applicable) and how the editor was applied.* |
| Authentication | *Describe any authentication procedures for each seed stock used or novel genotype generated. Describe any experiments used to assess the effect of a mutation and, where applicable, how potential secondary effects (e.g. second site T-DNA insertions, mosiacism, off-target gene editing) were examined.* |
